# Vitamin D status and its associations with bone mineral density, bone turnover markers, and parathyroid hormone in Chinese postmenopausal women with osteopenia and osteoporosis

**DOI:** 10.3389/fnut.2023.1307896

**Published:** 2024-01-10

**Authors:** Xi Chen, Li Shen, Chao Gao, Rou Weng, Yier Fan, Shuqin Xu, Zhenlin Zhang, Weiwei Hu

**Affiliations:** ^1^Department of Osteoporosis and Bone Disease, Shanghai Clinical Research Center of Bone Disease, Sixth People’s Hospital Affiliated to Shanghai Jiao Tong University School of Medicine, Shanghai, China; ^2^Clinical Research Center, Sixth People’s Hospital Affiliated to Shanghai Jiao Tong University School of Medicine, Shanghai, China

**Keywords:** vitamin D status, bone turnover markers, bone mineral density, osteopenia, osteoporosis, postmenopausal women

## Abstract

**Background:**

Vitamin D is a key factor in bone metabolism, yet vitamin D insufficiency and deficiency are prevalent among postmenopausal women, with potential repercussions on bone mineral density (BMD), bone turnover markers (BTMs), and parathyroid hormone (PTH). Nonetheless, the findings from existing studies exhibit inconsistency, and a notable gap exists in the availability of large-scale investigations.

**Methods:**

In this real-world study, 8,532 postmenopausal women over 50 years old with a diagnosis of osteopenia (50.9%) and osteoporosis (49.1%) at the first visit were enrolled in this study. Serum 25(OH)D level, PTH, osteocalcin (OC) and Beta-CrossLaps of type 1 collagen containing cross-linked C-telopeptide (β-CTX), were measured. BMD at all sites, including the lumbar spine, femoral neck, and total hip were obtained by dual-energy X-ray absorptiometry (DXA). The associations of serum 25(OH)D level with BMDs and BTMs were investigated using spearman correlation analysis and analysis of general linear model adjusted by age and body mass index.

**Results:**

The serum 25(OH)D level was 22.17 ± 9.75 ng/mL among all patients included in this study. For the osteopenia group, the serum 25(OH)D level was 22.40 ± 9.41 ng/mL, while for the osteoporosis group, it measured 21.93 ± 10.08 ng/mL. In the osteopenia group, the prevalence of vitamin D deficiency, insufficiency and sufficiency was 45.8, 34.6, and 19.6%, respectively, which was close to that of the osteoporosis group (47.4, 34.3, and 18.3%) (*p* = 0.202). Spearman correlation analysis unveiled negative associations between serum 25(OH)D concentrations and both BTMs and PTH within both the osteopenia and osteoporosis group. In the osteoporosis group, there were positive correlations between 25(OH)D levels and femoral neck BMD (*r* = 0.040, *p* = 0.010) and total hip BMD (*r* = 0.053, *p* = 0.001). Furthermore, we found that for the osteopenia group, greater vitamin D levels were associated with greater femoral neck BMD (*p* = 0.020) and total hip BMD (*p* = 0.008) and lower β-CTX (*p* < 0.001), OC (*p* < 0.001), and PTH (*p* < 0.001). The same trends were seen in osteoporosis patients (*p* < 0.05), and with greater lumbar spine BMD with higher levels of 25(OH)D (*p* = 0.009).

**Conclusion:**

This study showed high prevalence of vitamin D deficiency and insufficiency in Chinese postmenopausal women with osteopenia and osteoporosis and the relationships between vitamin D and BMD, BTMs and PTH. The results contribute to a more comprehensive understanding of how vitamin D may impact bone health.

## 1 Introduction

Vitamin D is a fat-soluble vitamin essential for bone metabolism, reducing the risk of rickets in children and osteomalacia in adults by improving intestinal calcium absorption (1). Vitamin D, including vitamin D_2_ and vitamin D_3_, is usually ingested by the body through exposure to sunlight or diet. Then, vitamin D converts to 25-hydroxyvitamin D [25(OH)D] by vitamin D-25-hydroxylase in the liver, and 25(OH)D is further converted to its active form, 1,25-dihydroxyvitamin D [1,25(OH)_2_D] in the kidneys by the enzyme 25-hydroxyvitamin D-1α-hydroxylase (2). Serum 25(OH)D is currently considered the most reliable marker for vitamin D status due to its long half-life in the circulation (3). Vitamin D deficiency (25(OH)D < 20 ng/mL) and insufficiency (20 ≤ 25(OH)D < 30 ng/mL) (4), are widespread globally and in China, especially among postmenopausal women (5, 6). Owing to estrogen deficiency, postmenopausal women have severe bone loss. Additionally, they are at high risk of vitamin D deficiency (7, 8). Kuchuk et al. conducted a study on 7,441 postmenopausal women with osteoporosis from 29 countries and found that the prevalence of 25(OH)D < 10, 10–20, 20–30, and >30 ng/mL was 5.9%, 29.4%, 43.5%, and 21.2%, respectively (9). In a nationwide, multicenter, cross-sectional study of 1,674 Chinese postmenopausal women, the prevalence of vitamin D insufficiency was 91.2% and of vitamin D deficiency was 61.3% (8). Nonetheless, it is noteworthy that comprehensive studies involving large sample sizes concerning the prevalence of vitamin D insufficiency and deficiency in China are currently limited in their availability.

Recently, there have been numerous studies investigating the effect of 25(OH)D on bone mineral density (BMD) in postmenopausal women (10–20), but the results are controversial. In description studies, some concluded that 25(OH)D was positively correlated with BMD at different sites, such as with femoral neck BMD (10, 11), with lumbar spine and femoral neck BMD (12, 13), and with all three of lumbar spine, femoral neck, and total hip BMD (14). However, there are some studies that suggest no correlation between 25(OH)D and BMD (15, 16). In randomized controlled trials, most studies of vitamin D supplementation for postmenopausal women have not been effective in increasing BMD (17, 18, 20). For example, Cooper et al. did not observe a significant increase in BMD at any site between the calcium supplementation group and the calcium plus vitamin D_2_ supplementation group (20). But Bislev et al. observed that although there was no difference in areal BMD (aBMD) measured by dual-energy X-ray absorptiometry (DXA), volumetric BMD (vBMD) (QCT scans) in the trochanter region and the femoral neck region was significantly higher in the vitamin D_3_ supplementation group than in the placebo group (19).

Bone turnover markers (BTMs) have been widely used to assess bone resorption and formation by measuring their concentration in blood and urine, including bone formation markers, such as osteocalcin (OC), and bone resorption markers, such as Beta-CrossLaps of type 1 collagen containing cross-linked C-telopeptide (β-CTX) (21). But, the relationship of 25(OH)D with BTMs is unclear. Some studies have suggested that 25(OH)D levels are negatively correlated with BTMs (including OC, β-CTX and N-terminal propeptide of type 1 collagen (P1NP)) (9, 18, 22–24). However, some studies have not found a significant correlation between 25(OH)D levels and BTMs (15, 19, 25–27). Parathyroid hormone (PTH) is also a hormone that plays an important role in calcium and phosphorus metabolism and can increase serum calcium and decrease serum phosphorus by stimulating the bones, kidney, and small intestine (28). Studies have shown that there is an inverse correlation between 25(OH)D and PTH in postmenopausal women (9, 22, 25, 26).

However, previous studies have suffered from the disadvantage of small sample sizes, and many focus only on postmenopausal women with osteoporosis, or severely low BMD (T-score ≤ −2.5), yet a much larger number of women have osteopenia, or moderately low BMD (−2.5 < T-score < −1.0) compared to osteoporosis (29). So, there is a need for large-scale studies to examine the relationship between 25(OH) D, BMD, and BTMs in postmenopausal women with osteopenia and osteoporosis. This deeper understanding is crucial for devising the most effective interventions. These varing results underscore the complex role of 25(OH)D in bone metabolism and highlight the imperative need for further investigation. Utilizing real-world data sourced from electronic health records (EHRs) of superior quality constitutes a more advantageous approach for addressing this particular research topic. In past decades, we have established a retrospective cohort of patients with osteopenia and osteoporosis from which we can conduct EHR research.

Therefore, in this real-world study, we aim to describe vitamin D status in postmenopausal women with osteopenia and osteoporosis in Shanghai, China, and to investigate the relationship between 25(OH)D and BMD, BTMs and PTH in this large sample population.

## 2 Materials and methods

### 2.1 Patients

The Shanghai Clinical Research Center of Bone Disease has treated a large number of patients with bone metabolic diseases, including osteopenia, osteoporosis, osteomalacia, hypophosphatemia, Paget’s disease and more. We have constructed a specialized database for osteopenia and osteoporosis from January 2012 to May 2020, and we included patients diagnosed with osteopenia and osteoporosis at their first visit and not taking anti-osteoporotic medication. Medical data, including anthropometrics, demographics, BMD, BTMs, as well as laboratory and diagnostic characteristics, were collected during the initial inpatient visit. This study is a large-sample cross-sectional study based on a real-world database and we obtained written approval from the Institutional Review Boards of Sixth People’s Hospital Affiliated to Shanghai Jiao Tong University School of Medicine. In our study, informed consent was not obtained from the patients due to the utilization of anonymized data extracted from EHRs.

The inclusion criteria of this study were: (1) age > 50 years and at least 1 year after menopause, which was defined as the absence of menstruation for at least 1 year; (2) osteopenia: BMD measured by DXA met −2.5 < T score < −1.0; (3) osteoporosis: T score ≤ −2.5 or had a fragility fracture of the hip or vertebrae or fragility fractures of the proximal humerus, pelvis or distal forearm with osteopenia; (4) not taking anti-osteoporotic medication, and no use of calcium or vitamin D in the last 6 months. Patients were excluded from this study when they met any of the following criteria: (1) a history of non-fragility fracture in the past 12 months; (2) malignancy; (3) secondary osteoporosis; (4) severe renal insufficiency (creatinine clearance ≤30 mL/min); (5) osteomalacia, hyperparathyroidism, hyperthyroidism and other metabolic bone diseases; (6) women who were in the premenopausal or perimenopausal phase; (7) Absence of BTMs or 25(OH)D or PTH examination records.

**Figure 1:**
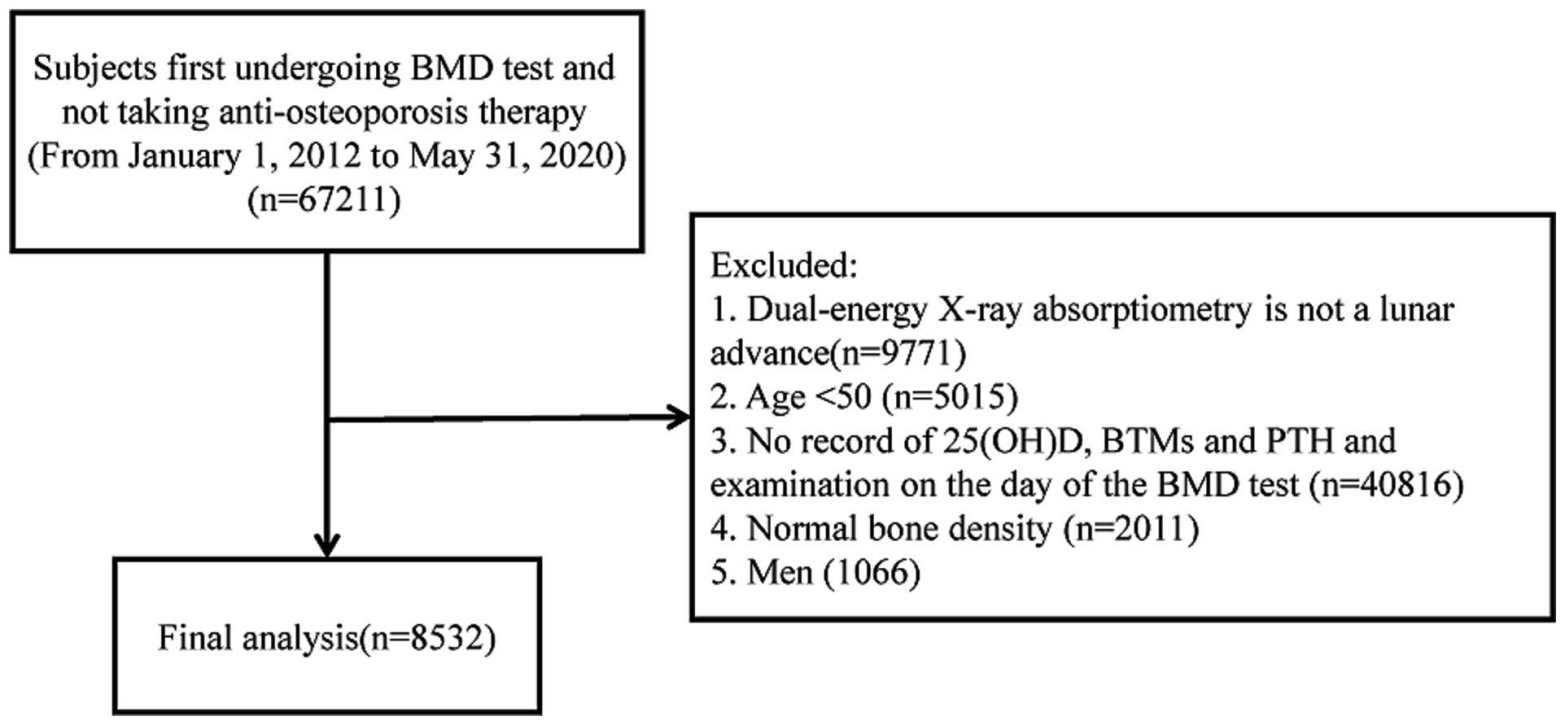
Flow chart of the study. BMD, bone mineral density; 25(OH)D, 25-hydroxyvitamin D; PTH, parathyroid hormone; BTM, bone turnover markers.

The final sample for analysis included 8,532 patients, including 4,339 with osteopenia and 4,193 with osteoporosis (see flow chart in Figure 1).

### 2.2 Biochemical measurements, BMD, and anthropometry

Blood samples were collected from patients after overnight fasting for serum levels of PTH, 25(OH)D, OC, β-CTX, and electrolytes, as well as liver and kidney functions. The above biochemical measurements were obtained using an automated Roche electrochemiluminescence system (Roche Diagnostic GmbH), following the manufacturer’s protocol. BMD of the lumbar spine, femoral neck and total hip were measured with a DXA (Prodigy Advance, GE Lunar Corporation, United States). For the patients with fractures or spondylarthrosis in the lumbar spine, BMD values at fracture and spondylarthrosis sites were excluded. The DXA scanner was calibrated daily with the coefficient of variability (CV) values for the DXA measurements – 1.39% at L1–4, 2.22% at the femoral neck, 0.70% at the total hip (30). BMD measurements of all patients were performed by specialized technicians. Strict quality control is carried out during BMD measurements.

Vitamin D deficiency is defined as 25(OH)D < 20 ng/mL, vitamin D insufficiency as 20 ≤ 25(OH)D < 30 ng/mL, and vitamin D sufficiency as 25(OH)D ≥ 30 ng/mL (31).

### 2.3 Statistical analysis

The main objective of this study was to investigate the correlation between 25(OH)D levels and BMD, BTMs, and PTH. According to the *α* of 0.05, when the sample size was 8,532, the correlation coefficient between 25(OH)D and femoral neck BMD, total hip BMD, β-CTX, OC and PTH was 0.049, 0.052, −0.238, −0.260, and −0.264 and the calculated power were all greater than 99%.

Normally distributed variables, skewed variables, and categorical variables were described using mean ± standard deviation, median (upper quartile, lower quartile), and frequency (percentage), respectively. Serum 25(OH)D levels were divided into three groups, deficiency (<20 ng/mL), insufficiency (20–30 ng/mL), and sufficiency (≥30 ng/mL). Comparisons between groups at different 25(OH)D levels were made using ANOVA or Kruskal–Wallis tests for continuous variables and chi-square tests for categorical variables. For *post hoc* tests, we used Bonferroni for multiplicity adjustment. Between-group comparisons of 25(OH)D levels between patients with osteopenia and osteoporosis were made using independent samples *t*-tests. Between-group comparisons of categorical variables between patients with osteopenia and osteoporosis were made using chi-square tests and Fisher exact method. Spearman correlation analysis was used to test the correlations between serum 25(OH)D levels and BMD, BTMs, and PTH. Coefficients were employed as measures to quantify the extent of the relationships between the variables.

To determine the association of serum 25(OH)D levels with BMD, BTMs and serum PTH, serum 25(OH)D level was entered as fixed factor, and BMD, BTMs or serum PTH as dependent factors for general linear model after adjustment for age and body mass index (BMI). *p* values for trends were calculated using the three groups of median values as quasi-continuous variables in the model.

Statistical significance was determined at a threshold of *p* < 0.05. All statistical analyses were performed using R software 4.3.0 (R Foundation for Statistical Computing, Vienna, Austria) and IBM SPSS (version 26.0; SPSS Inc., Chicago, IL).

## 3 Results

### 3.1 Demographic and clinical characteristics

This study included a total of 8,532 postmenopausal women, as shown in Figure 1. Among them, 4,339 (50.9%) participants were diagnosed with osteopenia, while 4,193 (49.1%) participants were identified with osteoporosis. General characteristics for the osteopenia group and osteoporosis group are shown in Supplementary Table S1. Significant statistical differences were observed in age, height, weight, BMI, BMD, and BTMs between the two groups (*p* < 0.05). Patients within the osteoporosis group were found to be older (*p* < 0.001) and exhibited lower BMI (*p* < 0.001), BMD at all measured sites (*p* < 0.001), and 25(OH)D levels (*p* = 0.028). Conversely, they presented higher levels of β-CTX (*p* < 0.001), OC (*p* < 0.001), and PTH (*p* = 0.014).

Subsequently, the subjects were divided into three serum 25(OH)D groups, deficiency <20 ng/mL, insufficiency 20–30 ng/mL and sufficiency ≥30 ng/mL. Subject characteristics of these groups are shown in Table 1. Most patients (46.6%, 3,977) had deficient 25(OH)D levels followed by 34.5% (*n* = 2,940) patients had insufficient 25(OH)D levels, and 1,615 patients (18.9%) possessed sufficient 25(OH)D levels. The distribution of osteoporosis did not exhibit any statistically significant distinctions among the three groups. There were statistically significant differences in age, weight, BMI, previous fractures, β-CTX, OC, PTH, serum calcium, phosphorus, creatinine, eGFR and BMD at all sites between patients with different 25(OH)D levels (*p* < 0.05). Mean age was significantly older in the sufficiency group than in the other groups. Mean weight was significantly higher in the deficiency group than in the other groups. Patients in the deficiency group had the highest β-CTX, OC, and PTH levels than the other groups.

**Table 1:** Baseline anthropometric and biochemical characteristics of participants according to different 25(OH)D levels.

Serum 25(OH)D level	All (*n* = 8,532)	Deficiency, <20 (*n* = 3,977)	Insufficiency, 20–30 (*n* = 2,940)	Sufficiency, ≥30 (*n* = 1,615)	*p*
Age (year)	67.68 ± 8.68	67.89 ± 8.86^a^	67.08 ± 8.42^b^	68.22 ± 8.62	**<0.001**
Height (cm)	153.75 ± 6.39	153.73 ± 6.47	153.72 ± 6.26	153.82 ± 6.44	0.869
Weight (kg)	55.30 ± 8.43	56.14 ± 8.49^a^^,^^c^	55.27 ± 8.29^b^	53.27 ± 8.22	**<0.001**
BMI (kg/m^2^)	23.40 ± 3.35	23.76 ± 3.40^a^^,^^c^	23.39 ± 3.23^b^	22.52 ± 3.31	**<0.001**
Lumbar spine 1–4 BMD (g/cm^2^)	0.86 ± 0.15	0.86 ± 0.14	0.85 ± 0.15^b^	0.87 ± 0.15	**0.037**
Femoral neck BMD (g/cm^2^)	0.69 ± 0.09	0.69 ± 0.09^a^	0.70 ± 0.09	0.69 ± 0.09	**<0.001**
Total hip BMD (g/cm^2^)	0.73 ± 0.11	0.73 ± 0.11^a^	0.74 ± 0.10	0.73 ± 0.11	**<0.001**
Osteoporosis (*n*, %)	4,193 (49.1)	1988 (50.0)	1,441 (49.0)	764 (47.3)	0.195
Previous fractures (*n*, %)	501 (5.9)	198 (5.0)^a^	202 (6.9)	101 (6.3)	**0.003**
β-CTX (ng/L)	467.0 (280.7,671.3)	529.9 (353.2,736.6)^a^^,^^c^	431.0 (253.6,612.2)^b^	357.8 (186.4,578.3)	**<0.001**
OC (ng/mL)	19.5 (14.4,25.8)	21.8 (16.1,28.7)^a^^,^^c^	18.5 (13.9,24.1)^b^	16.6 (12.0,22.2)	**<0.001**
PTH (ng/L)	40.8 (32.2,53.1)	45.2 (35.2,60.8)^a^^,^^c^	39.7 (31.2,49.3)^b^	36.3 (28.9,45.2)	**<0.001**
Serum Ca (mmol/L)	2.38 ± 0.21	2.38 ± 0.22	2.37 ± 0.23	2.37 ± 0.12	**0.047**
Serum P (mmol/L)	1.13 ± 0.17	1.12 ± 0.18^a^^,^^c^	1.14 ± 0.16	1.14 ± 0.16	**<0.001**
Serum creatinine (μmol/L)	58.0 (51.0,66.0)	58.0 (51.0,66.0)^c^	57.0 (51.0,66.0)^b^	59.0 (52.0,66.0)	**<0.001**
eGFR (ml/min)	72.03 ± 22.65	72.76 ± 23.69^c^	73.46 ± 22.63^b^	67.62 ± 19.27	**<0.001**
25(OH)D (ng/mL)	22.17 ± 9.75	14.36 ± 3.77^a^^,^^c^	24.31 ± 2.84^b^	37.51 ± 7.64	**<0.001**

Continuous variables are presented as mean ± SD or median (Q1, Q3). Categorical variables are presented as number (percent). BMI, body mass index; BMD, bone mineral density; β-CTX, Beta-CrossLaps of type 1 collagen containing cross-linked C-telopeptide; OC, osteocalcin; PTH, parathyroid hormone; Ca, calcium; P, phosphorus; eGFR, estimated glomerular filtration rate; 25(OH)D, 25-hydroxy vitamin D. Significant values (*p* < 0.05) are presented in bold.

a Statistically significant difference between the deficiency group compared to the insufficiency group.

b Statistically significant difference between the insufficiency group and the sufficiency group.

c Statistically significant difference between the deficiency group and the sufficiency group.

### 3.2 Prevalence of vitamin D deficiency, insufficiency, and sufficiency

As show in Table 2, the serum level of 25(OH)D was greater in patients with osteopenia (22.40 ± 9.41 ng/mL) than osteoporosis (21.93 ± 10.08 ng/mL) (*p* = 0.028). The prevalence of vitamin D deficiency, insufficiency and sufficiency were not different between patients with osteopenia and osteoporosis (*p* = 0.202).

**Table 2:** Distribution of 25(OH)D levels in postmenopausal women with osteopenia and osteoporosis.

	Osteopenia (*n* = 4,339)	Osteoporosis (*n* = 4,193)	*p*
25(OH)D (ng/mL)	22.40 ± 9.41	21.93 ± 10.08	**0.028**
25(OH)D (*n*, %)			0.202
Deficiency (<20 ng/mL)	1989 (45.8)	1988 (47.4)	
Insufficiency (20–30 ng/mL)	1,500 (34.6)	1,440 (34.3)	
Sufficiency (≥30 ng/mL)	850 (19.6)	765 (18.3)	

25(OH)D, 25-hydroxy vitamin D. Significant values (*p* < 0.05) are presented in bold.

To further understand the details of 25(OH)D distribution in postmenopausal women with osteopenia and osteoporosis, we stratified this population by age group, as is shown in Table 3. In the age groups 50–59, 60–69, 70–79, and ≥80, the median of 25(OH)D levels were 20.99 (15.50, 26.64) ng/mL, 20.58 (15.63, 27.37) ng/mL, 21.03 (14.74, 28.84) ng/mL, 20.04 (13.56, 26.71) ng/mL, respectively. There was statistically significant differences in the distribution of 25(OH)D levels across age groups (*p* = 0.001), and median 25(OH)D levels was significantly lower in the ≥80 age group than in the other age groups. Furthermore, in all age groups, it was the 25(OH)D deficiency group that had the highest percentage of patients, and the 25(OH)D sufficient group had the smallest percentage of patients.

**Table 3:** Distribution of 25(OH)D levels in postmenopausal women of different ages.

Age	*N*	All (*n* = 8,532)			
		25(OH)D ng/mL	Deficiency, <20 ng/mL	Insufficiency, 20–30 ng/mL	Sufficiency, ≥30 ng/mL
50–59	1,516	20.99 (15.50,26.64)	680 (44.9%)	578 (38.1%)^a^^,^^b^	258 (17.0%)^d^
60–69	3,789	20.58 (15.63,27.37)	1775 (46.8%)	1,352 (35.7%)^c^	662 (17.5%)^e^
70–79	2,274	21.03 (14.74,28.84)	1,051 (46.2%)	708 (31.2%)	515 (22.6%)
≥80	953	20.04 (13.56,26.71)^†^	471 (49.4%)	302 (31.7%)	180 (18.9%)
*p* value		**0.001** ^**#**^	**<0.001** ^*^		

Continuous variables are presented as median (Q1, Q3). Categorical variables are presented as number (percent). 25(OH)D, 25-hydroxy vitamin D. Significant values (*p* < 0.05) are presented in bold. #: Kruskal-Wallis test to compare the differences in 25(OH)D levels across age groups. ^†^: Post-hoc analysis showed that the difference in 25(OH)D levels between subjects in the ≥ 80 years old group and all other age groups was statistically significant.^*^: The Chi-square test to compare the deficiency of 25(OH)D levels in different groups.

a There is a statistically significant difference in the insufficiency proportation in the 50–59 age group compared to the 70–79 age group.

b There is a statistically significant difference in the insufficiency proportation in the 50–59 age group compared to the ≥80 age group.

c There is a statistically significant difference in the insufficiency proportation in the 60–69 age group compared to the ≥80 age group.

d There is a statistically significant difference in the sufficiency proportation in the 50–59 age group compared to the 70–79 age group.

e There is a statistically significant difference in the sufficiency proportation in the 60–69 age group compared to the 70–79 age group.

### 3.3 Relationships of vitamin D status with BMD in Chinese osteopenia and osteoporosis patients

This study used Spearman correlation analysis to examine the relationship between serum 25(OH)D levels and BMD (Table 4). The overall sample of postmenopausal women included in this study, including patients with osteopenia and patients with osteoporosis, a positive correlation was identified between 25(OH)D levels and lumbar spine 1–4 BMD (*r* = 0.023, *p* = 0.037), femoral neck BMD (*r* = 0.049, *p* < 0.001), and total hip BMD (*r* = 0.052, *p* < 0.001). In osteoporosis patients, serum 25(OH)D levels exhibited a positive correlation with femoral neck BMD (*r* = 0.040, *p* = 0.010) and total hip BMD (*r* = 0.053, *p* = 0.001).

**Table 4:** Spearman correlation analysis of serum 25(OH)D levels with BMD, BTMs and PTH in osteopenia and osteoporosis patients.

	All (*n* = 8,532)		Osteopenia (*n* = 4,339)		Osteoporosis (*n* = 4,193)	
	*r*	*P*	*r*	*P*	*r*	*P*
Lumbar spine 1–4 BMD (g/cm^2^)	0.023	**0.037**	−0.002	0.907	−0.012	0.437
Femoral neck BMD (g/cm^2^)	0.049	**<0.001**	0.025	0.101	0.040	**0.010**
Total hip BMD (g/cm^2^)	0.052	**<0.001**	0.023	0.124	0.053	**0.001**
β-CTX (ng/L)	−0.238	**<0.001**	−0.223	**<0.001**	−0.245	**<0.001**
OC (ng/mL)	−0.260	**<0.001**	−0.233	**<0.001**	−0.285	**<0.001**
PTH (ng/L)	−0.264	**<0.001**	−0.267	**<0.001**	−0.262	**<0.001**

BMD, bone mineral density; 25(OH)D, 25-hydroxyvitamin D; β-CTX, Beta-CrossLaps of type 1 collagen containing cross-linked C-telopeptide; OC, osteocalcin; PTH, parathyroid hormone; *r*, correlation coefficient. Spearman correlation analysis was used to the correlation between 25(OH)D levels and BMD, β-CTX, OC and PTH. Significant values (*p* < 0.05) are presented in bold.

**Figure 2:**
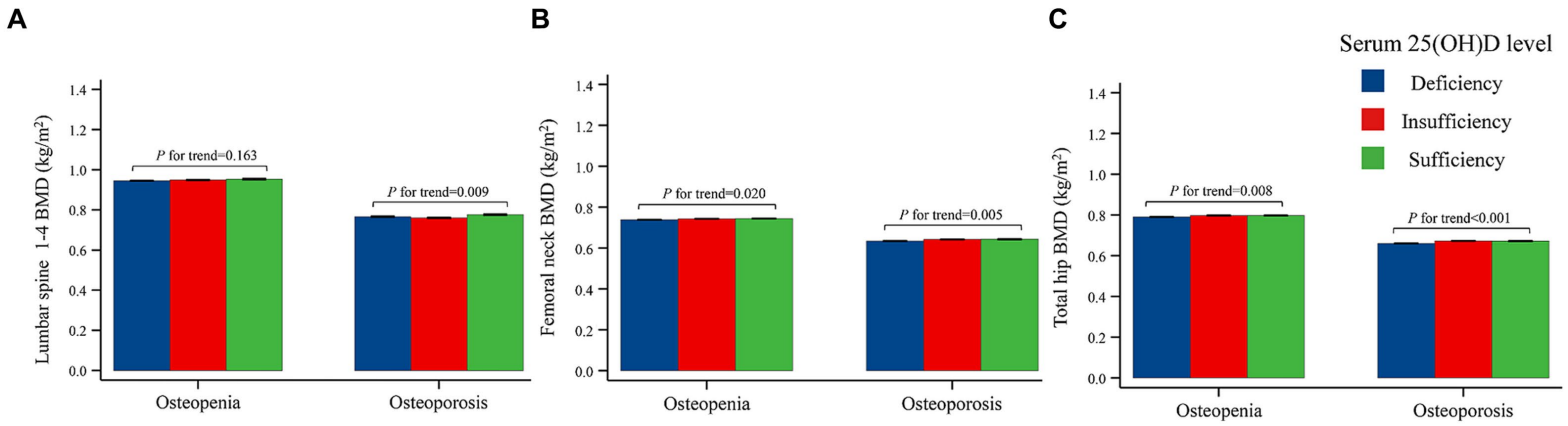
Bone mineral densities categorized 25(OH)D concentrations adjusted for age and BMI in osteopenia and osteoporosis patients. **(A)** Correlation of serum 25(OH)D and Lumbar spine 1–4 BMD. **(B)** Correlation of serum 25(OH)D and femoral neck BMD. **(C)** Correlation of serum 25(OH)D and total hip BMD. *p* values for trends were calculated using trend tests to investigate whether the levels of BMD, BTMs, and PTH were correlated with the 25(OH)D levels after divided the 25(OH)D levels into three groups (deficiency group, insufficiency group and sufficiency group). BMD, bone mineral density; 25(OH)D, 25-hydroxyvitamin D.

This study used general linear model to identify the associations between the 25(OH)D status and BMD, adjusted for age and BMI (Tables 5, 6 and Figure 2). It was observed that after adjusted for age and BMI and stratified by 25(OH)D level, for osteopenia patients, greater vitamin D levels were associated with greater femoral neck BMD (*p* = 0.020) and total hip BMD (*p* = 0.008). Similar trends were observed in osteoporosis patients (*p* < 0.05), and greater 25(OH)D levels was also associated with greater lumbar spine 1–4 BMD (*p* = 0.009).

**Table 5:** Serum bone turnover markers, serum parathyroid hormone concentrations, and bone mineral densities categorized 25(OH)D concentrations adjusted for age and BMI in osteopenia patients.

Serum 25(OH)D level (ng/mL)	Deficiency, <20 (*n* = 1989)	Insufficiency, 20–30 (*n* = 1,500)	Sufficiency, ≥30 (*n* = 850)	*p* for trend
Lumbar spine 1–4 BMD (g/cm^2^)	0.945 (0.002)	0.949 (0.003)	0.953 (0.004)	0.163
Femoral neck BMD (g/cm^2^)	0.738 (0.002)	0.743 (0.002)	0.744 (0.002)	**0.020**
Total hip BMD (g/cm^2^)	0.790 (0.002)	0.797 (0.002)	0.797 (0.003)	**0.008**
β-CTX (ng/L)	484.73 (9.14)	418.36 (10.49)	380.70 (14.07)	**<0.001**
OC (ng/mL)	21.83 (0.19)	18.53 (0.22)	17.10 (0.29)	**<0.001**
PTH (ng/L)	53.07 (0.67)	41.59 (0.77)	39.56 (1.03)	**<0.001**

PTH, parathyroid hormone; BMD, bone mineral density; 25(OH)D, 25-hydroxyvitamin D; β-CTX, Beta-CrossLaps of type 1 collagen containing cross-linked C-telopeptide; OC, osteocalcin. Values are adjusted mean (SE) after weighting all variables with adjustment for age and BMI. *p* values for trends were calculated using the three sets of median values as quasi-continuous variables in the general linear model.

**Table 6:** Serum bone turnover markers, serum parathyroid hormone concentrations and bone mineral densities categorized 25(OH)D concentrations adjusted for age and BMI in osteoporosis patients.

Serum 25(OH)D level (ng/mL)	Deficiency, <20 (*n* = 1988)	Insufficiency, 20–30 (*n* = 1,440)	Sufficiency, ≥30 (*n* = 765)	*p* for trend
Lumbar spine 1–4 BMD (g/cm^2^)	0.766 (0.003)	0.760 (0.003)	0.776 (0.004)	**0.009**
Femoral neck BMD (g/cm^2^)	0.634 (0.002)	0.642 (0.002)	0.643 (0.003)	**0.005**
Total hip BMD (g/cm^2^)	0.660 (0.002)	0.673 (0.002)	0.672 (0.003)	**<0.001**
β-CTX (ng/L)	654.98 (8.78)	530.42 (10.31)	467.70 (14.22)	**<0.001**
OC (ng/mL)	28.88 (0.39)	21.90 (0.45)	18.82 (0.62)	**<0.001**
PTH (ng/L)	62.73 (1.08)	43.64 (1.27)	39.36 (1.75)	**<0.001**

PTH, parathyroid hormone; BMD, bone mineral density; 25(OH)D, 25-hydroxyvitamin D; β-CTX, Beta-CrossLaps of type 1 collagen containing cross-linked C-telopeptide; OC, osteocalcin. Values are adjusted mean (SE) after weighting all variables with adjustment for age and BMI. *p* values for trends were calculated using the three sets of median values as quasi-continuous variables in the general linear model.

### 3.4 Relationships of vitamin D status with BTMs and PTH in Chinese osteopenia and osteoporosis patients

As shown in Table 4, for both osteopenia and osteoporosis patients, the 25(OH)D level displayed a negative correlation with β-CTX (*r* = −0.223, *p* < 0.001; *r* = −0.245, *p* < 0.001), OC (*r* = −0.233, *p* < 0.001; *r* = −0.285, *p* < 0.001), and PTH (*r* = −0.267, *p* < 0.001; *r* = −0.262, *p* < 0.001).

**Figure 3:**
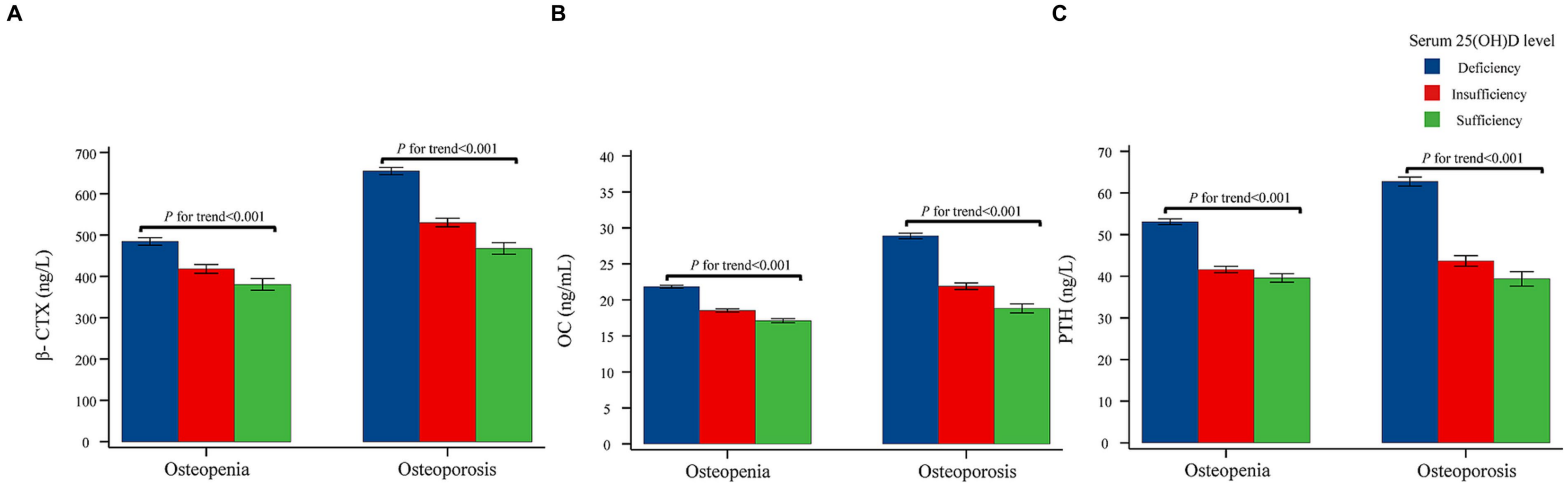
Serum turnover markers and serum parathyroid hormone concentrations categorized 25(OH)D concentrations adjusted for age and BMI in osteopenia and osteoporosis patients. **(A)** Correlation of serum 25(OH)D and β-CTX. **(B)** Correlation of serum 25(OH)D and OC. **(C)** Correlation of serum 25(OH)D and PTH. *p* values for trends were calculated using trend tests to investigate whether the levels of BMD, BTMs, and PTH were correlated with the 25(OH)D levels after divided the 25(OH)D levels into three groups (deficiency group, insufficiency group and sufficiency group). 25(OH)D, 25-hydroxyvitamin D; β-CTX, Beta-CrossLaps of type 1 collagen containing cross-linked C-telopeptide; OC, osteocalcin; PTH, parathyroid hormone.

As shown in Tables 5, 6 and Figure 3, after adjusted for age and BMI and stratified by 25(OH)D level, for both osteopenia and osteoporosis patients, greater 25(OH)D level was associated with decreasing levels of β-CTX (*p* < 0.001), OC (*p* < 0.001), and PTH (*p* < 0.001).

## 4 Discussion

In this real-world study, we observed positive correlations between serum 25(OH)D levels and BMD, as well as negative correlations with BTMs and PTH within osteopenia and osteoporosis patients. Our study represents the most extensive investigation to date that specifically focuses on assessing vitamin D status and elucidating the associations between 25(OH)D levels and parameters such as BMD, BTMs, and PTH concentrations among postmenopausal women diagnosed with osteopenia and osteoporosis in Shanghai, China. Additionally, our study provides comprehensive insights into these conditions within osteopenia and osteoporosis populations, thereby raising awareness about the crucial role of vitamin D in these conditions.

Firstly, we investigated vitamin D status in Shanghai postmenopausal women with osteopenia and osteoporosis. Notably, prior studies have consistently highlighted the issue of suboptimal vitamin D levels among the Chinese population. A multicenter cross-sectional study involving 1,674 Chinese postmenopausal women by Xie et al. showed that the serum 25(OH)D was 18.0 ± 8.4 ng/mL, and 91.2% of this population had vitamin D < 30 ng/mL and 61.3% had vitamin D < 20 ng/mL (8). In a study of 5,067 postmenopausal women in the Shanghai suburban area, Gao et al. found that the serum 25(OH)D levels in this population were 24.5 ± 13.2 ng/mL, 37.7% had vitamin D insufficiency and 30.5% had vitamin D deficiency (22). Zhang et al.’s study of 101 healthy postmenopausal women in the Shanghai community found that the mean value of serum 25(OH)D was 17.09 ng/mL, vitamin D insufficiency accounting for 30% and vitamin D deficiency accounting for 68% (32). In our extensive study, which encompassed 8,532 postmenopausal women in Shanghai, we observed an average 25(OH)D level of 22.17 ± 9.75 ng/mL. Furthermore, we identified that 34.5% of the sample had vitamin D insufficiency, and 46.6% had vitamin D deficiency. And we also provided a detailed description of vitamin D distribution in osteopenia, osteoporosis and different age groups, as shown in Tables 2, 3. These findings are consistent with prior research in China (8, 22, 32), collectively underscoring the widespread issue of insufficient vitamin D levels and the high prevalence of vitamin D insufficiency and deficiency within the Chinese population.

Subsequently, we used spearman correlation analysis and analysis of general linear model to explore the associations of vitamin D with BMD in the Chinese population. Numerous studies have explored the relationship between vitamin D and BMD in postmenopausal women (9, 10, 12, 13, 16, 22, 25, 26, 33–38), but the results are controversial. For example, some studies have suggested a positive correlation between vitamin D and BMD at different sites, including femoral neck BMD (10, 25, 33, 34), total hip BMD (22), and both femoral neck and lumbar spine BMD (12, 13, 35, 36, 39). Nevertheless, other research findings have indicated no significant association between vitamin D and BMD (16, 26, 37). Reasons for the difference results may include fewer patients being included in these studies and not categorizing women according to BMD. To address these limitations, we enrolled a total of 8,532 postmenopausal women in our study, which is the largest sample size study in China that we are aware of, and we categorized the patients into osteopenia and osteoporosis according to BMD. After adjusted for age and BMI, we finally came to the conclusion: for patients with osteoporosis, greater vitamin D levels were associated with greater BMD at all sites; for patients with osteopenia, greater vitamin D levels were associated with greater femoral neck BMD and total hip BMD. However, it is worth noting that both in this study and in previous studies that concluded a positive correlation between vitamin D and BMD, the correlation coefficients between vitamin D and BMD were generally less than 0.35 (9–14, 34–36). These relatively low correlation coefficients implies that it is difficult to achieve a significant increase in BMD in postmenopausal women through vitamin D supplementation. In postmenopausal women, the goal of maintaining adequate vitamin D status may be to maintain existing bone mass or slow inevitable bone loss.

In addition, the relationship between vitamin D and BTMs is still unclear. Some studies have suggested a negative correlation between vitamin D levels and BTMs (including OC, β-CTX and P1NP) (9, 18, 22–24). Nonetheless, certain studies have proposed that there is no significant correlation between vitamin D levels and BTMs (15, 19, 25–27). A 1 year randomized controlled trial conducted by Grimnes et al. examined 297 postmenopausal women with a BMD T-score < −2.0 supplemented with either a high-dose vitamin D3 (6,500 IU vitamin D_3_/day) or a standard dose (800 IU vitamin D_3_/day). Both groups were found to have decreased levels of BTMs, namely P1NP and β-CTX (18). Similarly, we observed a negative correlation between vitamin D and BTMs, including β-CTX and OC, in both women with osteopenia and osteoporosis. In addition, our investigation revealed a negative correlation between vitamin D and PTH, similar to other studies (9, 22, 26). In case of vitamin D deficiency, the concentration of the active vitamin D metabolite, 1,25(OH)_2_D, may decrease, and then PTH levels increase, stimulating synthesis of 1,25(OH)_2_D in the kidney. The increased serum PTH stimulates bone turnover, leading to bone loss (40). Postmenopausal women have active bone turnover due to estrogen deficiency (41), indicated by BTMs (21). Appropriate vitamin D supplementation in postmenopausal women with vitamin D insufficiency or deficiency may help to maintain low BTMs and PTH and may help reduce bone loss, though further longitudinal research is needed. For vitamin D deficiency patients, the recommended supplementation varies from region to region (3, 42). Chinese guidelines recommend a loading dose of 300,000 IU, such as 50,000 IU capsules once a week for 6 weeks, followed by maintenance therapy with 800–2,000 IU daily (3).

Our study has several obvious advantages. First, the main strength in this study lies in its substantial sample size, affording ample statistical power for the rigorous examination of the relationship between 25(OH)D levels and BMD and BTMs. Second, we categorized the postmenopausal women based on BMD values, and focused on patients with osteopenia and osteoporosis, providing support for research in postmenopausal women with osteopenia. Third, all BMD, BTMs and biochemical tests in this study were performed using standardized procedures, with professional quality control staff for quality control of the operations. Other strengths of this study include the relatively rich data in real-world clinical practice which can reflect clinical realities. Some limitations should be acknowledged. Primarily, it is imperative to underscore that this study constitutes a single-center retrospective investigation conducted exclusively in Shanghai. It is noteworthy that specific sociodemographic attributes pertaining to healthcare system participants in this locale may exhibit variations when compared to those in other geographical regions of China. Second, though all participants were enrolled at their first visit, which means they did not accept any prior treatment in our hospital, some participants may have been treated at other hospitals before. Furthermore, it is important to highlight that some conditions, such as smoking, exercise, daily calcium intake, education level and family income, were found to be absent from the EHR dataset.

## 5 Conclusion

In conclusion, this study showed high prevalence of vitamin D deficiency and insufficiency in Chinese postmenopausal women with osteopenia and osteoporosis. The relationships demonstrated between vitamin D and BMD, BTMs, and PTH may contribute to a more comprehensive understanding of how vitamin D can impact bone health.

## Data availability statement

The original contributions presented in the study are included in the article/Supplementary Material, further inquiries can be directed to the corresponding authors.

## Ethics statement

We obtained written approval from the Institutional Review Boards of Sixth People’s Hospital Affiliated to Shanghai Jiao Tong University School of Medicine. Informed consent was not obtained from the patients due to the utilization of anonymized data extracted from electronic health records.

## Author contributions

XC: Conceptualization, Methodology, Writing – original draft, Writing – review & editing. LS: Conceptualization, Formal analysis, Methodology, Writing – original draft, Writing – review & editing. CG: Data curation, Writing-review & editing. RW: Data curation, Writing – review & editing. YF: Data curation, Writing – review & editing. SX: Supervision, Writing – review & editing. ZZ: Supervision, Writing – review & editing. WH: Supervision, Writing – review & editing.
