# Supplementary material for: Vitamin D status and its associations with bone mineral density, bone turnover markers, and parathyroid hormone in Chinese postmenopausal women with osteopenia and osteoporosis
Source: Front Nutr. 2024 Jan 10;10:1307896. doi: 10.3389/fnut.2023.1307896 (PMC10806182; doi:10.3389/fnut.2023.1307896)
Supplement: Supplementary file 1 [file Table_1.docx]

Supplementary Material

# Supplementary Figures and Tables

## Supplementary Tables

Supplementary Table 1. Baseline anthropometric and biochemical characteristics of participants in the osteopenia group and osteoporosis group

| Serum 25(OH)D level | All  (n=8532) | Osteopenia  (n=4339) | Osteoporosis  (n=4193) | *P* value |
| --- | --- | --- | --- | --- |
| Age (year) | 67.68±8.68 | 66.54±8.39 | 68.86±8.81 | **<0.001** |
| Height (cm) | 153.75±6.39 | 155.14±5.89 | 152.30±6.58 | **<0.001** |
| Weight (kg) | 55.30±8.43 | 57.95±7.96 | 52.55±8.02 | **<0.001** |
| BMI (kg/m^2^) | 23.40±3.35 | 24.11±3.29 | 22.67±3.26 | **<0.001** |
| Lumbar spine 1-4 BMD (kg/m^2^) | 0.86±0.15 | 0.95±0.11 | 0.77±0.12 | **<0.001** |
| Femoral neck BMD (kg/m^2^) | 0.69±0.09 | 0.74±0.07 | 0.64±0.09 | **<0.001** |
| Total hip BMD (kg/m^2^) | 0.73±0.11 | 0.79±0.08 | 0.67±0.09 | **<0.001** |
| β-CTX (ng/L) | 467.0(280.7,671.3) | 422.7(255.2,598.9) | 517.9(310.0,749.1) | **<0.001** |
| OC (ng/mL) | 19.5(14.4,25.8) | 18.5(13.7,24.1) | 20.9(15.4,28.0) | **<0.001** |
| PTH (ng/L) | 40.8(32.2,53.1) | 40.5(32.3,52.2) | 41.2(32.1,54.2) | **0.014** |
| 25(OH)D (ng/mL) | 22.17±9.75 | 22.40±9.41 | 21.93±10.08 | **0.028** |
| 25(OH)D level, n (%) |  |  |  | 0.202 |
| Deficiency | 3977(46.6) | 1989(45.8) | 1988(47.4) |  |
| Insufficiency | 2940(34.5) | 1500(34.6) | 1440(34.3) |  |
| Sufficiency | 1615(18.9) | 850(19.6) | 765(18.3) |  |

Note: Continuous variables are presented as mean ± SD or median (Q1, Q3). Categorical variables are presented as number (percent). Abbreviations: BMI, body mass index; BMD, bone mineral density; β-CTX, Beta-CrossLaps of type 1 collagen containing cross-linked C-telopeptide; OC, osteocalcin; PTH, parathyroid hormone; 25(OH)D, 25-hydroxyvitamin D. Significant values (*P*<0.05) are presented in bold.
